# *ZmILI1* regulates leaf angle by directly affecting *liguleless1* expression in maize

**DOI:** 10.1111/pbi.13255

**Published:** 2019-10-01

**Authors:** Zhenzhen Ren, Liancheng Wu, Lixia Ku, Huitao Wang, Haixia Zeng, Huihui Su, Li Wei, Dandan Dou, Huafeng Liu, Yingying Cao, Dongling Zhang, Shengbo Han, Yanhui Chen

**Affiliations:** ^1^ College of Agronomy, Synergetic Innovation Center of Henan Grain Crops and National Key, Laboratory of Wheat and Maize Crop Science Henan Agricultural University Zhengzhou China

**Keywords:** *qLA2*, *liguleless1*, fine mapping, feedback loop, leaf angle, maize, promoter, signalling pathway

Crop yield is determined by the capacity of the plant canopy to capture light energy and store nitrogen (Sinclair and Sheehy, 1999), which in turn depends mostly on the photosynthetic rate *per se* and canopy architecture of the plant. Leaf angle (LA), which is defined as the inclination between the leaf blade midrib and the vertical culm, is one of the most important parameters describing the canopy architecture (Anderson and Denmead, 1969). It is a complex trait, and several related quantitative trait loci (QTLs) have been identified; however, only two of these have been functionally characterized in maize (Ku *et al.*, 2012; Tian *et al.*, 2019). A few LA maize mutants have been found, and their corresponding genes have been cloned, such as *liguleless1* (*LG1*), which is involved in cell division and differentiation. It is a mutant known to affect LA in maize (Moreno *et al.*, 1997), and the mutation results in an absence of ligule or auricle, thus leading to considerably more upright leaves than the wild type. The *ZmLG1* mutation induced by RNA‐guided Cas9 reduces the LA of the overall canopy (Li *et al.*, 2017; Wang *et al.*, 2019). Further analysis of the progeny indicated that the mutation was largely heritable and the hybrids carrying the mutation had an average LA that was 50% smaller than the wild type; this translated to an increased plant density of 90 000 plants/ha, and an increase in the net photosynthesis and yield by 16% and 2%, respectively (Li *et al.*, 2017). However, the molecular mechanisms by which these genes control LA in maize need further exploration.

To identify new regulators of LA in maize, we conducted a QTL analysis of LA in the F_2:3_ population derived from crossing the compact inbred line Yu82 and the expanded inbred line Yu87‐1. A major QTL, *qLA2*, was identified, and it explained 9.42% of the phenotypic variance (Ku *et al.*, 2012). The QTL does not overlap with the reported genes controlling LA, indicating that QTL, *qLA2*, is a novel genetic component in LA regulation. We developed a near‐isogenic line of Yu82 (named Yu82‐NIL) for *qLA2* through repeated backcrossing and molecular‐assisted selection by using Yu82 as the receptor parent and D132 as the donor parent. The LA of Yu82‐NIL was found to be 17.46° greater than that of Yu82, indicating that *qLA2* indeed contains an important locus controlling LA in maize (Figure 1a).

**Figure 1:**
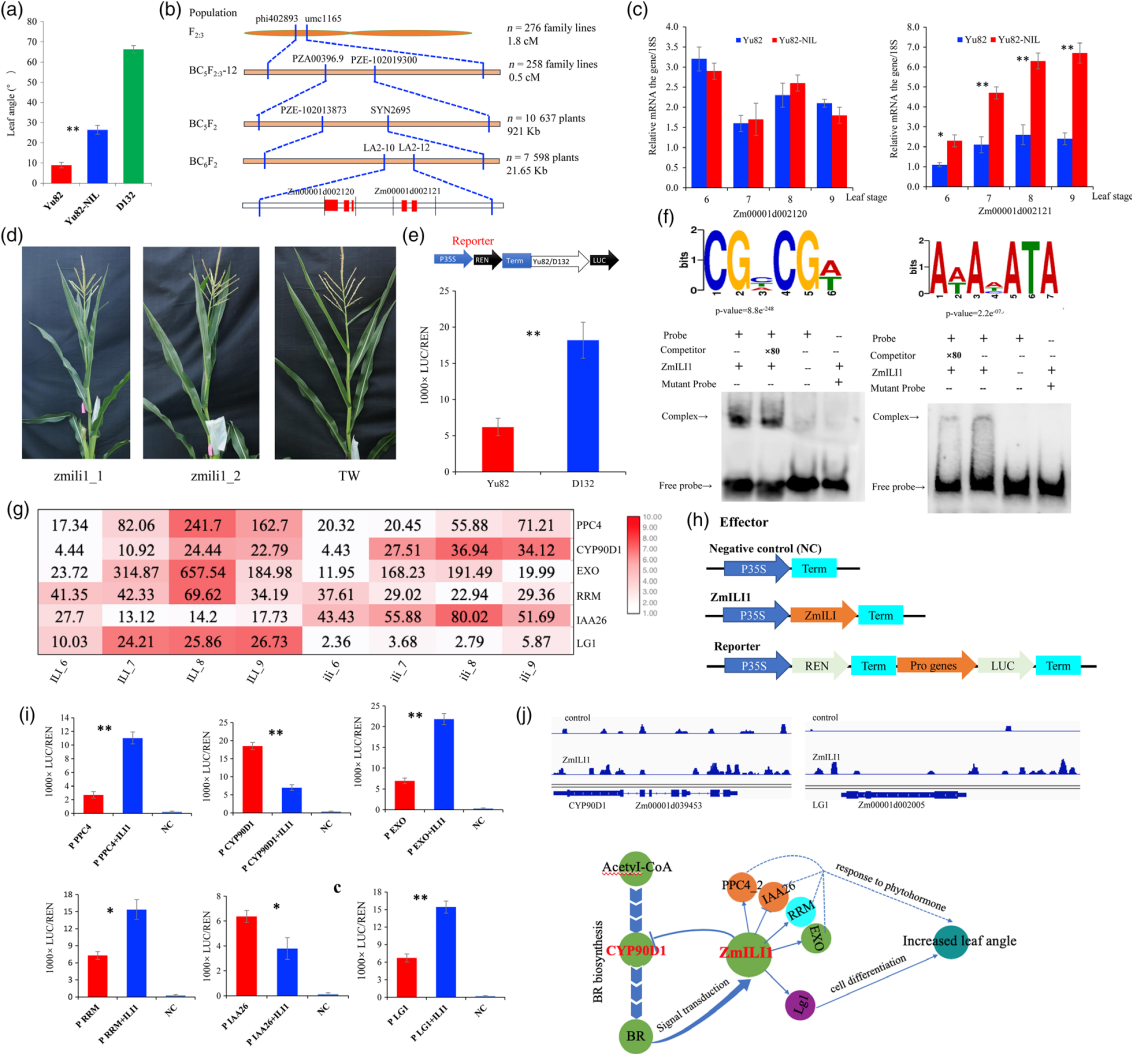
*ZmILI1* functional mechanism for LA formation in maize. (a) The mean LA statistics for all leaves above the uppermost ear (*n* = 10, **P < 0.01). (b) Sequential fine mapping of *ZmILI1*. The red square boxes represent exons in the genes. (c) The expression profiles of the two candidate genes in the limited region (*P < 0.05, **P < 0.01). (d) CRISPR knockout lines of Zmili1 showing reduced LA. (e) The upper panel shows the promoter region of a diagram of the construct used for the promoter activity assay. The lower panel shows the constructs that were introduced into *N benthamiana* leaves; the expression levels were determined using the Luc activity assay (**P < 0.01). (f) *ZmILI1* binding to the CGNCGN and ANANATA core motifs (upper panel); EMSA results confirming the in vitro binding of *ZmILI1* to CGTCGA and AAACATA (lower panel). (g) Heat map showing the differentially expressed genes bound by *ZmILI1*. (h) The 35S:REN‐Pro PR:LUC reporter constructs were transiently expressed in *N. benthamiana* leaves together with control vector or 35S:*ZmILI1* effector. (i) The LUC/REN ratio represents the relative activity of the gene promoters (*P < 0.05, **P < 0.01). (j) Two‐target gene binding peaks for the positive control genes are shown in the Integrated Genome Brower (upper panel); a schematic model for LA formation in maize (lower panel). The arrows between the genes stand for promotion or activation, and the T bars between the genes indicate suppression. The orange circles represent auxin‐responsive genes; the cyan circle represents cytokinin‐responsive gene; the green circles represent brassinosteroid‐responsive genes; and the purple circles represent cell differentiation genes.

To clone the gene for *qLA2*, we mapped *qLA2* using BC_5_F_2_ and BC_6_F_2_ populations (with a total of 18 235 individuals) and developed markers, and narrowed down *qLA2* to a genomic region between the markers LA2‐9 and LA2‐11 (~21.65 kb in length) covered by a single BAC clone (AC202705) on chromosome 2 (Figure 1b). Of the two predicted genes in the region, Zm00001d002120 was not differentially expressed in the two lines, but Zm00001d002121 was consistently expressed at a significantly higher level in leaves of Yu82‐NIL than in those of Yu82, at the 6‐ to 9‐leaf stage of development (Figure 1c). Thus, we viewed Zm00001d002121 as the candidate gene for *qLA2*. The gene is an ortholog of *OsILI1* reportedly a positive regulator that plays an important role in LA in rice (Zhang *et al.*, 2009); therefore, we named it *ZmILI1*. It is a basic helix–loop–helix leucine zipper family transcription factor that harbours only the helix–loop–helix domain but lacks the basic region.

To verify that Zm00001d002121 corresponds to *ZmILI1*, we generated knockout mutants of Zm00001d002121 using CRISPR/cas9 technology; these mutants showed decreased LA (Figure 1d**)**. To test the possible regulatory role of this promoter in expression control, we conducted a dual‐luciferase (Luc) transient assay with Yu82 and D132 promoters in *Nicotiana benthamiana* leaves. The results showed that the Luc reporter gene driven by the D132 promoter exhibited significantly higher expression than the reporter gene from the Yu82 promoter (Figure 1e**)**, suggesting that promoter variations could explain the variations in its expression level and further affect its functionality.

To investigate the regulatory mechanism mediated by *ZmILI1*, we performed DAP‐Seq assays to uncover the genes directly targeted by *ZmILI1*. We predicted *ZmILI1*‐binding sites using MACS2 software with *P*‐value < 0.05 (based on a Poisson distribution comparing the *ZmILI1* sample and the control) and identified 6249 peaks across the whole genome through *ZmILI1‐*binding motifs CGNCGN and ANANATA (Figure 1f). Of the *ZmILI1‐*binding sites, 48.3% (3018 peaks) were located in the genic regions containing the genes, as well as 5 kb upstream and downstream of the start and stop codons, respectively. EMSA further confirmed the *ZmILI1*‐binding motifs CGNCGN and ANANATA. The 3018 peaks correspond to 2893 genes; more specifically, *ZmILI1* binds to upstream regions of 1062 genes. To further demonstrate the expression level of the genes as putative targets directly modulated by *ZmILI1*, RNA‐Seq data were generated from wild type and zmili1 lines. In 1062 putative target genes directly modulated by *ZmILI1* in upstream regions, differential expression of six genes seemed to be responsible for LA; four of these genes were up‐regulated in *ZmILI1* (Figure 1g). The up/down‐regulated genes are mainly involved in responses to auxins, cytokinins, brassinosteroids (BR) and cell differentiation.

To understand whether *ZmILI1* functions as a transcriptional factor of the identified up/down‐regulated genes, we performed Luc transient transcriptional activity assays in *N. benthamiana* leaves with *ZmILI1* driven by the 35S promoter as an effecter and Luc as the reporter gene (Figure 1h). The results showed that *ZmILI1* specifically repressed the expression of Luc from the Zm00001d039513 (IAA26) and Zm00001d039453 (CYP90D1) promoters and increased the expression from Zm00001d002005 (LG1), Zm00001d047447 (PPC4), Zm00001d013271 (RRM) and Zm00001d052206 (EXO) promoters, indicating that these genes are target genes of *ZmILI1* **(**Figure 1i). Thus, in our study, we found that *ZmILI1* binds to the *ZmLG1* promoter (−1868 bp; Figure 1j), which is a new regulatory relationship unreported in other species. In addition, *ZmILI1* binds to the CYP90D1 promoter (−897 bp; Figure 1j), which is involved in distinct BR‐biosynthetic steps and likely participates in the oxidative C‐3 epimerization of BRs (Kim *et al.*, 2005; Tang *et al.*, 2010). It follows that biosynthesized BRs, via cytochrome P450‐catalysed oxidative reactions mediated by CYP90D1, could be delivered to BR receptor BAK1 to bind to nuclear transcription factors BZR1 and BZR2/BES1 (Tang *et al.*, 2010) and ultimately bind ZmILI; this binding would repress CYP90D1 (Figure 1j) and thus affect BR biosynthesis. Furthermore, the signal transduction could form a new negative feedback loop in maize that has not been reported in other species.

In summary, our results demonstrate that *ZmILI1* is a central regulator of multiple signalling and developmental pathways related to LA formation during maize development, acting in both direct and indirect ways to affect the patterns of gene transcription. A better understanding of *ZmILI1* regulatory pathways will provide new insights into useful information for the elucidation of the molecular mechanisms underlying maize LA formation in the future.

## Conflict of interest

The authors declare no conflicts of interest.

## Author contributions

L.K., L. Wu., L. Wei. and Z.R. performed fine mapping of *ZmILI1* and generated and characterized the transgenic plants. Z.R., H.L., D.D., H.Z., D.Z., H.W., S.H. Y. Cao and H.S. conducted molecular biology experiments. Y. Chen, L.K., L.W. and Z.R. designed the experiments and wrote the manuscript.
